# Prevalence of depressive symptoms among older adults who reported medical cost as a barrier to seeking health care: findings from a nationally representative sample

**DOI:** 10.1186/s12877-019-1203-2

**Published:** 2019-07-18

**Authors:** Vinay K. Cheruvu, Edward T. Chiyaka

**Affiliations:** College of Public Health, Kent State University, 320 Lowry Hall, 750 Hilltop Drive, Kent, OH 44242 USA

**Keywords:** Medical cost, Out-of-pocket expenses, Depression, Current depressive symptoms, BRFSS

## Abstract

**Background:**

Older adults aged 65 and over will make up more than 20% of U.S. residents by 2030, and in 2050, this population will reach 83.7 million. Depression among older adults is a major public health concern projected to be the second leading cause of disease burden. Despite having Medicare, and other employer supplements, the burden of out of pocket healthcare expenses may be an important predictor of depression. The current study aims to investigate whether delay in seeing a doctor when needed but could not because of medical cost is significantly associated with symptoms of current depression in older adults.

**Methods:**

Cross-sectional data from the 2011 Behavioral Risk Factor Surveillance System (BFRSS) from 12 states and Puerto Rico were used for this study (*n* = 24,018).

**Results:**

The prevalence of symptoms of current depression among older adults who reported medical cost as a barrier to seeking health care was significantly higher (17.8%) when compared to older adults who reported medical cost not being a barrier to seeking health care (5.5%). Older adults who reported medical cost as a barrier to seeking health care were more likely to report current depressive symptoms compared to their counterparts [Adjusted Odds Ratio (AOR): 2.2 [95% CI: 1.5–3.3]).

**Conclusions:**

Older adults (≥ 65 years of age) who experience the burden of medical cost for health care are significantly more likely to report symptoms of depression. Health care professionals and policymakers should consider effective interventions to improve access to health care among older adults.

**Electronic supplementary material:**

The online version of this article (10.1186/s12877-019-1203-2) contains supplementary material, which is available to authorized users.

## Background

The rapid aging of the United States population is due, in part; to the increase in life expectancy and the aging of the post-World War II baby boom generation [1]. By 2030, older adults aged 65 and over will make up more than 20% of U.S. residents, and by 2050, this population will reach 83.7 million, almost double the 2012 estimated population of 43.1 million [1, 2]. Older adults are disproportionately affected by chronic conditions with approximately 60% living with at least one chronic condition and 42% with at least two chronic conditions [3]. Access to health services is essential for prevention and management of chronic illnesses to minimize the disease burden and associated health care costs. The overarching goal of Healthy People 2020 is to improve the health, function, and quality of life of older adults, which includes an objective to increase the proportion of older adults who are up to date on a core set of clinical preventive health services for maintaining quality of life and overall wellness [4].

Depression is a serious problem and a major public health concern among older adults [5, 6]. The prevalence of depression among community dwelling adults aged 65 and older is estimated to be between 5 and 10% and is projected to be the second leading cause of disease burden in this population by the year 2020 [7, 8]. Risk factors for depression such as chronic diseases [9], disability [10, 11], lack of social support, and socio-economic status [12–14], have been well documented. Given that aging is associated with multiple chronic health conditions and limited financial resources for disease management, and despite the availability of Medicare, Medicaid and employer supplements [15–19], the burden of out of pocket healthcare expenses may be an important predictor of depression.

Although several studies have documented the risk factors for depression in the elderly [9, 10, 12–14], and while others have examined the impact of depression on healthcare costs [20–22], to the best of our knowledge, out of pocket healthcare expenses (e.g., delay in seeing a doctor when needed but could not because of medical cost) as a predictor of current depressive symptomatology in a nationally representative sample of community-dwelling older adults in the U.S. has not been documented yet. Previous reported research on the association between financial strain and depressive symptoms among older adults focused on financial strain measured by a 4-item scale and was not specific to financial strain due to the burden of out of pocket healthcare expenses [23, 24]. As such, the purpose of this study is to:

1. estimate the prevalence of current depressive symptoms in a sample of community dwelling older adults (≥ 65 years of age) who reported a delay in seeing a doctor when needed but could not because of medical cost,
2. investigate whether delay in seeing a doctor when needed but could not because of medical cost is significantly associated with symptoms of current depression in the elderly after accounting for possible potential confounders, and.
3. describe the socio-demographic characteristics of older adults who are more likely to report delay in seeing a doctor when needed but could not because of medical cost.

This current study is motivated by “The theory of cost-sharing” framework. Cost-sharing is any kind of out-of-pocket expenses made by individuals for health care services. It may result in individuals’ delaying a follow-up health care visit or completely wait out a health problem [25, 26]. Therefore, we sought to understand the relationship between cost-sharing, as defined by medical cost as a barrier to seeking health care, on symptoms of current depression which is an important health outcome.

## Methods

### General study design

The Behavioral Risk Factor and Surveillance System (BRFSS) is a federally funded telephone survey designed and conducted annually by the Centers for Disease Control and Prevention (CDC) in collaboration with state health departments in all 50 states, Washington, DC; Puerto Rico; the U.S. Virgin Islands; and Guam. The survey collects data on health conditions, preventive health practices, and risk behaviors of the adults selected [27]. All BRFSS questionnaires, data, and reports are available at http://www.cdc.gov/brfss/. Data for this study were obtained from 12 states (Kansas, Louisiana, Maine, Mississippi, Nebraska, Nevada, New Hampshire, New Jersey, New Mexico, New York, Ohio, and Oregon) and Puerto Rico that administered the ‘Anxiety and Depression’ optional module in the 2011 BRFSS data. The weighted response rates ranged from 33.4 to 61.7%.

### Delay in seeing a doctor due to cost: primary exposure of interest

To determine if cost is a barrier to seeking health care among older adults, responses to the following question: “*Was there a time in the past 12 months when you needed to see a doctor but could not because of cost*? ” were used to create a binary exposure variable (Yes / No).

### Current depressive symptoms: primary outcome of interest

Current depressive symptomatology is defined based on the responses to the eight-item Patient Health Questionnaire (PHQ-8) depression scale [28]. The scores for each item, which range from 0 to 3, are summed to produce a total score between 0 and 24 points. Symptoms of current depression is defined as PHQ-8 score ≥ 10 [25]. The PHQ-8 consists of eight of the nine DSM-IV criteria for depressive disorders [29, 30].

### Covariates of interest

Socio-demographic variables: age, gender (male, female), race/ethnicity (White non-Hispanic, African American non-Hispanic, Hispanic, Others), marital status (married, unmarried), education (less than college, college graduate), and employment (employed, unemployed); Health indicators: general health (“*Excellent* / *Very good / Good”* or “*Fair / Poor”*), use of special equipment due to a health problem (Yes / No), smoking (Yes / No), and number of chronic conditions other than current depression; and Health care indicators: having a health plan (Yes / No), having a primary care provider (Yes / No), and having an annual checkup (Yes / No), were considered as covariates of interest in this study.

#### Statistical analyses

**Fig. 1:**
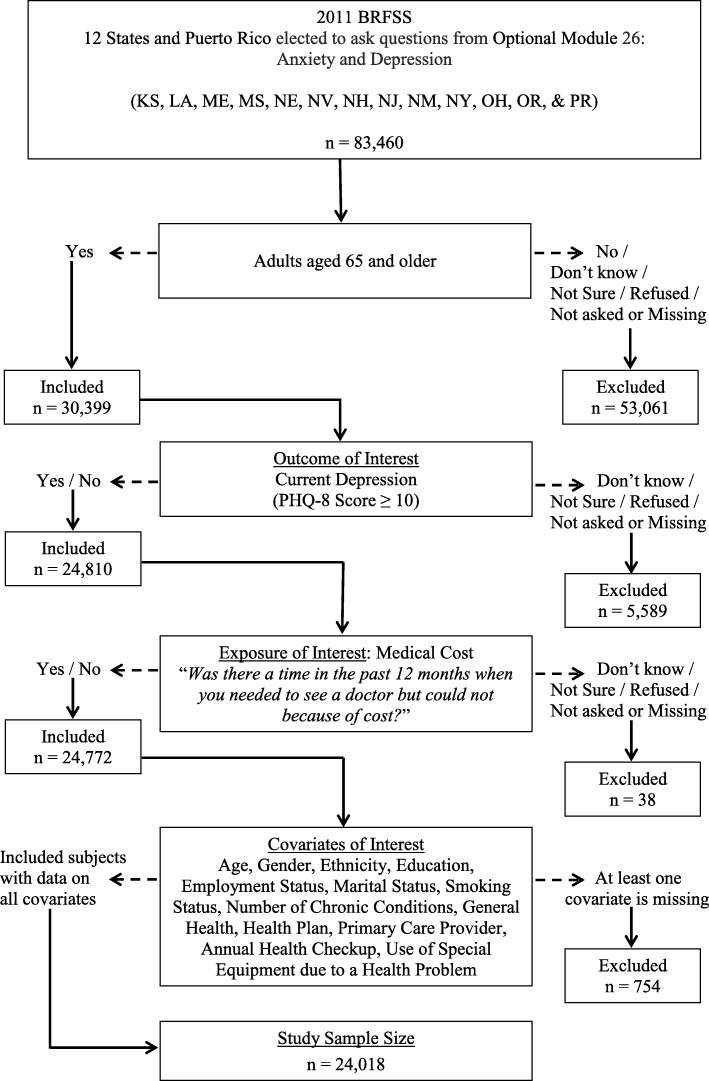
Flowchart of Study Population and Sample Size

Sampling weights provided in the 2011 BRFSS public-use data that adjust for unequal selection probabilities, survey non-response, and oversampling were used to account for the complex sampling design and to obtain population-based estimates that reflect U.S. non-institutionalized individuals. In order to describe the characteristics of the study population, weighted prevalence estimates, and corresponding 95% confidence intervals (CI) were computed based on the sample of individuals (*n* = 24,018) with complete data on all variables considered in this study (Fig. 1). Logistic regression models were used to examine if delay in seeing a doctor when needed but could not because of medical cost is significantly associated with symptoms of current depression in the elderly, adjusting for potential confounders and other covariates.

All analyses were conducted in SAS 9.3 (SAS Institute, Cary, NC, USA) using SAS survey procedures (PROC SURVEYFREQ, PROC SURVEYMEANS, PROC SURVEYLOGISTIC) to account for the complex sampling design.

## Results

### Sample characteristics

In 2011, the prevalence of current depressive symptoms among U.S. older adults from the 12 states and Puerto Rico was 6.1% (95% CI: 5.3–7.0%). About 6 % (5.7, 95% CI: 4.9–6.6%) reported medical cost as a barrier to seeking health care when needed in the past 12 months. Table 1 describes the overall characteristics of older adults, the weighted prevalence of reporting medical cost as a barrier to seeking health care and the weighted prevalence of current depressive symptoms along with the corresponding 95% confidence intervals. The average age of older adults at the time of survey was 74.1 years [95% CI: 73.9–74.4]. The majority of these adults were females (56.3%), White Non-Hispanic (77.5%), and more than three-fourth of them were either retired or unable to work. A little less than half of the older adults were unmarried (48.3%), and had more than high school education (44.6%). A little over one-fourth of the older adults reported “fair/poor” general health (28.8%), almost 80% of them had at least one chronic condition other than current depression, were predominantly non-smokers (91.2%), and did not use any special equipment due to a health problem (81.0%). The majority of older adults in the 12 states and Puerto Rico reported having a health plan (97.9%), having a primary care provider (94.7%), and having an annual health checkup (87.1%).

### Significant sub-groups of older adults with medical cost as a barrier to seeking health care

Among the different covariates examined for significant differences in the proportion of older adults who reported medical cost as a barrier to seeking health care when needed in the past 12 months, the following sub-groups of individuals were significantly higher in proportion to report medical cost as a barrier to seeking health care compared to some of the older adults who reported medical cost not being a barrier to seeking health care: *i)* Hispanics vs. White non-Hispanics (11.7% vs. 4.3%); *ii)* Black non-Hispanics vs. White non-Hispanics (10.1% vs. 4.3%); *iii)* older adults with no college education vs. those with some college education (7.2% vs. 3.9%); *iv)* those who reported “Fair/Poor” general health vs. those with “Excellent/Very Good/Good” general health (10.4% vs. 3.8%); *v)* those with 3 or more chronic conditions other than current depression vs. those with no chronic conditions (8.2% vs. 3.8%); *vi)* smokers vs non-smokers (11.3% vs. 5.2%); *vii)* those who reported use of special equipment due to a health condition vs. those who did not use (10.0% vs. 4.7%); *viii)* those with no health plan vs. those with a health plan (27.3% vs. 5.2%); *ix)* those with no primary care provider vs. those with a primary care provider (11.8% vs. 5.4%); and *x)* those who reported not having an annual health checkup vs. those who did (11.4% vs. 4.9%). There was no significant difference in the average age of older adults who reported medical cost as a barrier to seeking health care when compared to those who did not report medical cost as a barrier (73.0 years vs. 74.2 years).

### Significant sub-groups of older adults with current depressive symptoms

Females were significantly higher in proportion to report symptoms of current depression compared to males (7.4% vs. 4.6%). Among the sub-groups of individuals who reported medical cost as a barrier to seeking health care, the following also reported symptoms of current depression: *i)* Hispanics vs. White non-Hispanics (11.0% vs. 5.6%); *ii)* those who reported “Fair/Poor” general health vs. those with “Excellent/Very Good/Good” general health (14.3% vs. 2.8%); *iii)* those with 3 or more chronic conditions other than current depression vs. those with no chronic conditions (11.5% vs. 2.8%); i*v)* smokers vs non-smokers (10.8% vs. 5.7%); and *v)* those who reported use of special equipment due to a health condition vs. those who did not use (15.5% vs. 4.0%). Interestingly, older adults with a health plan were significantly higher in proportion for symptoms of current depression compared to those without a health plan (6.2% vs. 3.3%). Average age between individuals with and without symptoms of current depression was not significant (73.8 years vs. 74.1 years).

### Model based odds ratios for symptoms of current depression

The prevalence of symptoms of current depression among older adults who reported medical cost as a barrier to seeking health care was significantly higher [17.8, 95% CI: (11.4–21.1%)] when compared to older adults who reported medical cost not being a barrier to seeking health care [5.5, 95% CI: (4.7–6.2%)]. After adjusting for all covariates of interest, older adults who reported medical cost as a barrier to seeking health care were more than twice likely to report current depressive symptoms compared to older adults who reported medical cost not being a barrier to seeking health care (Adjusted Odds Ratio (AOR): 2.2 [95% CI: 1.5–3.3]) (Table 2). Additional file 1: Table S1 provides additional details on possible potential confounders we adjusted for in determining the association between medical cost as a barrier to seeking health care and current depressive symptoms in older adults.

### Predictors of reporting medical cost as a barrier to seeking health care

According to the adjusted logistic regression model results, gender (females: AOR: 1.52 [95% CI: 1.07–2.17]), ethnicity (Black non-Hispanics: AOR: 1.79 [95% CI: 1.09–2.96]; Hispanics: AOR: 2.48 [95% CI: 1.53–4.02]), education (less than college education: AOR: 1.49 [95% CI: 1.05–2.10]), general health (“Fair / Poor”: AOR: 2.12 [95% CI: 1.42–3.17]), smoking status (smokers: AOR: 1.72 [95% CI: 1.07–2.76]), having a health plan (No: AOR: 5.44 [95% CI: 2.67–11.09]), getting an annual checkup (No: AOR: 2.82 [95% CI: 1.91–4.17]), and use of special equipment due to a health problem (Yes: AOR: 1.64 [95% CI: 1.13–2.39]) were significant predictors of reporting medical cost as a barrier to seeking health care (Table 3).

## Discussion

### Study overview

Broadly speaking, studies on depression in the elderly have focused either on its risk factors or associated increases in health care costs and utilization. The current study is unique as it represents the first study of its kind to report the prevalence of symptoms of current depression in a sample of community dwelling older adults (≥ 65 years of age) in the U.S. who self-reported medical cost as a barrier to seeking health care. In addition, this study examined the association between self-reported medical cost as a barrier to seeking health care and current depressive symptomatology and identified the socio-demographic characteristics of older adults who had a significantly higher likelihood to report medical cost as a barrier to seeking health care. The findings from this study highlight the need for effective interventions to address the barriers to seeking health care due to the burden of out-of-pocket medical cost on the individual, particularly for those with multiple chronic conditions. This may help minimize the risk for current depression in older adults.

### Main findings

Based on data from the 2011 BRFSS, the prevalence of symptoms of current depression in a sample of community dwelling older adults (≥ 65 years of age) in the U.S. was 6.1%. This prevalence is higher than previously reported based on data from 2006 BRFSS using similar criteria (score ≥ 10 on PHQ-8) for current depressive symptoms [28]. The prevalence of current depressive symptomatology was significantly higher (17.8%) among older adults who reported medical cost as a barrier to seeking health care when compared to older adults who did not report medical cost as a barrier (5.5%). After adjusting for all covariates, we found that older adults who reported medical cost as a barrier to seeking health care were twice more likely to report current depressive symptoms compared to those who did not report medical cost as a barrier to seeking health care. In addition, we also found that older adults with the following *socio-demographics*: females, Black non-Hispanics, Hispanics, and high school or less education; *health indicators*: “Fair/Poor” general health, smokers, and use of special equipment due to a health problem; and *health care indicators*: not having a health plan, and no annual checkup, were significantly more likely to report medical cost as a barrier to seeking health care. This finding is consistent with prior studies that have identified socio-demographic characteristics associated with out-of-pocket medical cost [15, 31, 32].

### Association between medical cost as a barrier to seeking health care and symptoms of current depression

One of the most common barriers to seeking health care is out-of-pocket medical cost [33]. In particular, among older adults with chronic conditions, the burden of out-of-pocket medical cost is a major concern [34–36]. In this current study, though out-of-pocket health care expenses was not measured, consistent with previous studies [19, 37–39], we found that the proportion of older adults who reported medical cost as a barrier to seeking health care increased with the number of chronic conditions (Table 1). A plausible explanation could be that a significant proportion of medical cost may have to do with out-of-pocket expenses for medications and prescription drugs often required to manage chronic conditions [34–36, 39].

**Table 1:** Characteristics of Adults Aged 65 and Older (*n* = 24,018)

	Unweighted n	Overall Mean or Proportion (95% CI)		Yes, Medical Cost a Barrier to Health Care^a^ Mean or Proportion (95% CI)		Current Depressive Symptoms (PHQ-8 Score ≥ 10) Mean or Proportion (95% CI)	
Socio-demographics							
Age in years	24018	74.1	(73.9–74.4)	73.0	(72.1–74.0)	73.8	(72.2–75.4)
Gender							
Male	8457	43.7	(42.0–45.4)	4.7	(3.5–5.8)	4.6	(3.6–5.6)
Female	15561	56.3	(54.6–57.9)	6.5	(5.3–7.7)	7.4	(6.1–8.6)
Ethnicity							
White Non-Hispanic	20135	77.5	(76.0–79.0)	4.3	(3.5–5.2)	5.6	(4.7–6.4)
Black Non-Hispanic	1479	7.7	(6.7–8.7)	10.1	(6.6–13.6)	5.1	(2.9–7.3)
Hispanic	1785	11.9	(10.8–13.1)	11.7	(8.0–15.3)	11.0	(7.4–14.6)
Others^b^	619	2.9	(2.2–3.5)	6.3	(3.7–8.9)	4.8	(1.8–7.9)
Marital Status							
Married	11330	51.7	(50.1–53.4)	4.7	(3.6–5.8)	5.0	(4.0–6.0)
Unmarried^c^	12688	48.3	(46.6–49.9)	6.8	(5.5–8.1)	7.4	(6.0–8.8)
Education							
Elementary/Some High School/High School Graduate	11359	55.4	(53.8–57.0)	7.2	(5.9–8.5)	7.0	(5.7–8.3)
Some College or Technical School/College graduate	12659	44.6	(43.0–46.2)	3.9	(2.9–4.8)	5.1	(4.1–6.1)
Employment							
Employed	3520	13.6	(12.4–14.7)	4.9	(2.9–6.8)	5.4	(2.3–8.5)
Unemployed^d^	20498	86.4	(85.3–87.6)	5.9	(4.9–6.8)	6.3	(5.4–7.1)
Health Indicators							
General Health							
Excellent/Very Good/Good	17903	71.2	(69.6–72.7)	3.8	(3.0–4.7)	2.8	(2.1–3.6)
Fair/Poor	6115	28.8	(27.3–30.4)	10.4	(8.4–12.4)	14.3	(12.1–16.6)
Number of Chronic Conditions							
0	5123	20.9	(19.6–22.2)	3.8	(2.4–5.2)	2.8	(1.6–3.9)
1	7673	30.7	(29.2–32.2)	5.1	(3.8–6.4)	4.1	(2.6–5.6)
2	5719	24.4	(23.0–25.9)	5.7	(3.9–7.5)	6.4	(4.7–8.1)
≥ 3	5503	23.9	(22.4–25.4)	8.2	(6.0–10.4)	11.5	(9.4–13.7)
Smoking							
Yes	2103	8.8	(7.8–9.7)	11.3	(6.1–16.4)	10.8	(6.5–15.0)
No	21915	91.2	(90.3–92.2)	5.2	(4.4–5.9)	5.7	(5.0–6.5)
Use of Special Equipment due to a Health Problem							
Yes	4531	19.0	(17.7–20.2)	10.0	(7.4–12.4)	15.5	(12.8–18.1)
No	19487	81.0	(79.8–82.3)	4.7	(3.9–5.6)	4.0	(3.2–4.8)
Health Care Indicators							
Have a Health Plan							
Yes	23626	97.9	(97.2–98.5)	5.2	(4.4–6.0)	6.2	(5.4–7.1)
No	382	2.1	(1.5–2.8)	27.3	(13.1–41.5)	3.3	(1.5–5.1)
Have a Primary Care Provider							
Yes	22726	94.7	(93.9–95.5)	5.4	(4.5–6.2)	6.2	(5.3–7.0)
No	1292	5.3	(4.5–6.1)	11.8	(6.9–16.6)	6.0	(3.1–8.7)
Annual Health Checkup							
Yes	20255	87.1	(86.0–88.1)	4.9	(4.1–5.7)	6.1	(5.2–6.9)
No	3763	12.9	(11.8–14.0)	11.4	(7.8–15.1)	6.6	(3.8–9.5)

^a^ Was there a time in the past 12 months when you needed to see a doctor but could not because of cost?

^b ^Includes non-Hispanic Asian, Native Hawaiian or Other Pacific Islander, American Indian or Alaskan Native only, Multiracial and other race only

^c ^Includes Divorced, Widowed, Separated, Never Married, and Member of an unmarried couple

^d ^Includes Homemaker, Student, Retired, Unable to Work, and Out of work

**Table 2:** Prevalence of and Estimated Odds Ratio (95% CI) for Current Depressive Symptoms (PHQ-8 Score ≥ 10)

	Unweighted n	% (95% CI)	UOR (95% CI)	AOR (95% CI)
Was there a time in the past 12 months when you needed to see a doctor but could not because of cost?				
Yes	1062	17.8 (11.4–24.1)	3.7 (2.4–5.9)	2.2 (1.5–3.3)
No	22956	5.5 (4.7–6.2)	Reference	Reference

%: Prevalence, *UOR* Unadjusted Odds Ratio

*AOR* Adjusted Odds Ratio. Adjusted for age, gender, ethnicity, marital status, education, employment, number of chronic conditions, smoking, general health status, having a health plan, having a primary care provider, annual health checkup, and use of special equipment due to a health problem

**Table 3:** Predictors of Reporting Medical Cost as a Barrier to Seeking Health Care among Adults Aged 65 and Older (*n* = 24,018)

	Unadjusted Odds Ratio (UOR) and 95% CI for Medical Cost as a Barrier to Seeking Health Care		Adjusted Odds Ratio (AOR) and 95% CI for Medical Cost as a Barrier to Seeking Health Care	
Socio-demographics				
Age in years	0.98	(0.96–1.00)	0.97	(0.95–1.00)
Gender				
Male	Reference		Reference	
Female	**1.43**	**(1.04–1.97)**	**1.52**	**(1.07–2.17)**
Ethnicity				
White Non-Hispanic	Reference		Reference	
Black Non-Hispanic	**2.48**	**(1.60–3.83)**	**1.79**	**(1.09–2.96)**
Hispanic	**2.91**	**(1.94–4.36)**	**2.48**	**(1.54–4.02)**
Others^a^	1.48	(0.89–2.47)	1.37	(0.82–2.30)
Marital Status				
Married	Reference		Reference	
Unmarried^b^	**1.46**	**(1.07–2.01)**	1.10	(0.77–1.56)
Education				
Some College or Technical School/College Graduate	Reference		Reference	
Elementary/Some High School/High School Graduate	**1.92**	**(1.39–2.65)**	**1.49**	**(1.05–2.10)**
Employment				
Employed	Reference		Reference	
Unemployed^c^	1.21	(0.77–1.92)	0.92	(0.60–1.41)
Health Indicators				
General Health				
Excellent / Very Good / Good	Reference		Reference	
Fair / Poor	**2.92**	**(2.12–4.01)**	**2.12**	**(1.42–3.17)**
Number of Chronic Conditions				
0	Reference		Reference	
1	1.35	(0.85–2.16)	1.36	(0.81–2.28)
2	1.52	(0.92–2.54)	1.35	(0.70–2.61)
≥ 3	**2.24**	**(1.38–3.64)**	1.68	(0.92–3.07)
Smoking				
No	Reference		Reference	
Yes	**2.33**	**(1.36–3.99)**	**1.72**	**(1.07–2.76)**
Use of Special Equipment due to a Health Problem				
No	Reference		Reference	
Yes	**2.21**	**(1.58–3.09)**	**1.64**	**(1.13–2.39)**
Health Care Indicators				
Have a Health Plan				
Yes	Reference		Reference	
No	**6.79**	**(3.34–13.82)**	**5.44**	**(2.67–11.09)**
Have a Primary Care Provider				
Yes	Reference		Reference	
No	**2.34**	**(1.44–3.83))**	1.17	(0.72–1.88)
Annual Health Checkup				
Yes	Reference		Reference	
No	**2.53**	**(1.70–3.76)**	**2.83**	**(1.91–4.18)**

^a^Includes non-Hispanic Asian, Native Hawaiian or other Pacific Islander, American Indian or Alaskan Native only, Multiracial and other race only

^b^Includes Divorced, Widowed, Separated, Never Married, and Member of an unmarried couple

^c^Includes Homemaker, Student, Retired, Unable to Work, and Out of work

Statistical signifiance at 5% alpha is indicated by odds ratios in bold

Empirical evidence presented in the current study suggests that the burden of out-of-pocket medical cost is significantly associated with symptoms of current depression, thus, taking a step further in our understanding of the consequences of out-of-pocket medical cost (cost-sharing) burden among older adults. This finding is consistent with previous studies which reported the association between financial strain in general and symptoms of depression among older adults [23, 24].

Several studies have reported that lack of health care coverage or type of coverage (e.g., Medicare only) would lead to high financial burden [19, 36, 40]. This finding was consistent in our study with 27.3% reporting medical cost as a barrier for seeking health care among those without a health plan compared to 5.2% reporting the same among those with a health plan. However, despite having a health plan, the unadjusted prevalence of symptoms of current depression among older adults who reported medical cost as a barrier for seeking health care was 18.8% [95% CI: 11.8–25.7%]. Whereas among older adults without a health plan, the unadjusted prevalence of symptoms of current depression among those who reported medical cost as a barrier for seeking health care was 8.6% [95% CI: 2.2–15.0%]. This could be because older adults with a health plan are more likely to utilize health care and as a result might have to sustain more out-of-pocket expenses compared to those without a health plan.

While we don’t have specific information about the type of health plan, to understand the extent to which having a health plan mitigates the association between the burden of out-of-pocket medical cost and the likelihood to report current depression after controlling for all socio-demographic and health related factors, we examined the magnitude of this association stratified by health plan status. Among older adults who reported having a health plan, after adjusting for possible potential confounders, older adults who reported medical cost as a barrier for seeking health care were more than twice as likely to report current depression compared to those who reported medical cost not being a barrier for seeking health care [AOR: 2.2 [95% CI: 1.5–3.4]); whereas among those who reported not having a health plan, the magnitude of this association was much augmented [AOR: 7.3 [95% CI: 2.2–22.4]). This finding suggests that the burden of out-of-pocket medical cost is significantly associated with symptoms of current depression though the magnitude of this association could be mitigated due to having a health plan. As the prevalence of individuals with persistently high medical cost burden is likely to surge in the future [34], policies to reduce the burden of out-of-pocket medical cost among older adults may help address depression to some extent among the elderly and thereby reduce health care costs associated with depression.

### Strengths and limitations

Although this study documents, using a nationally representative sample, a significant association between the individuals’ burden of medical cost for health care and symptoms of depression among older adults, these findings are subject to limitations:

- The cross-sectional nature of the BRFSS data precludes us from drawing any causal relationship between the burden of medical cost and symptoms of current depression.
- BRFSS data are based on self-report and therefore may be subject to recall-bias for certain types of responses.
- Analysis of data in this study did not consider social and emotional support system among older adults. It is likely that social isolation in older adults might be a contributing factor to individual’s mental health [41, 42]. In addition, the analysis did not account for annual income; approximately 1 out of 7 older adults reported as employed during the time of the survey.
- Information about having a health plan was binary (Yes / No). Information about the type of health plan (e.g., types of insurance coverage) would have shed more light into the relationship between the burden of medical cost for health care and symptoms of current depression.
- Finally, since the data used in the current study are from 12 states in the 2011 BRFSS survey and is limited to non-institutionalized older adults, the findings may not be generalizable to all older adults in the U.S.

Despite these limitations, this study documents an important finding that has policy implications. To the best of our knowledge, this is the first study to report a significant association between individuals’ burden of medical cost for health care and symptoms of depression.

## Conclusions

In conclusion, findings from this study suggest that older adults (≥ 65 years of age) who experience the burden of medical cost for health care are significantly more likely to report symptoms of depression. Having a health plan might help mitigate the association between burden of medical cost for health care and symptoms of depression. Health care professionals and policymakers should consider effective interventions to improve access to health care among older adults. Such efforts may help address the mental health concerns such as depression among the elderly and thereby reduce the cost burden associated with it and improve health outcomes. Further research is necessary to confirm the findings from this study and to understand how older adults manage their comorbid conditions which puts on them a huge burden of out-of-pocket medical cost to deal with.

## Additional file

Additional file 1: **Table S1.** Estimated Odds Ratio (95% CI) for Current Depressive Symptoms (PHQ-8 Score ≥ 10) among Adults Aged 65 and Older (*n* = 24,018). (DOCX 20 kb)

## Data Availability

Datasets can be obtained from the corresponding author on reasonable request.

## Abbreviations

AOR: Adjusted Odds Ratio
BRFSS: Behavioral Risk Factors Surveillance System
CDC: Centers for Disease Control and Prevention
U.S: United States
UOR: Unadjusted Odds Ratio
