# Supplementary material for: Prevalence of depressive symptoms among older adults who reported medical cost as a barrier to seeking health care: findings from a nationally representative sample
Source: BMC Geriatr. 2019 Jul 18;19:192. doi: 10.1186/s12877-019-1203-2 (PMC6639933; doi:10.1186/s12877-019-1203-2)
Supplement: Supplementary file 1 — Table S1. Estimated Odds Ratio (95% CI) for Current Depressive Symptoms (PHQ-8 Score ≥ 10) among Adults Aged 65 and Older (n = 24,018). (DOCX 20 kb) [file 12877_2019_1203_MOESM1_ESM.docx]

| Additional file 1 Table S1. Estimated Odds Ratio (95% CI) for Current Depressive Symptoms (PHQ-8 Score ≥ 10) among Adults Aged 65 and Older (n = 24,018) | | | |
| --- | --- | --- | --- |
|  | **Adjusted Odds Ratio (AOR) and 95% CI for Current Depressive Symptoms**  **(PHQ-8 Score ≥ 10)** | |  |
| **Primary Exposure of Interest** |  |  |  |
| Was there a time in the past 12 months when you needed to see a doctor but could not because of cost? |  |  |  |
| No | Reference | |  |
| Yes | **2.23** | **(1.49 – 3.33)** |  |
|  |  |  |  |
| **Socio-demographics** |  |  |  |
| Age in years | 0.98 | (0.94 – 1.02) |  |
| Gender |  |  |  |
| Male | Reference | |  |
| Female | **1.56** | **(1.12 – 2.18)** |  |
| Ethnicity |  |  |  |
| White Non-Hispanic | Reference | |  |
| Black Non-Hispanic | 0.61 | (0.35 – 1.06) |  |
| Hispanic | 1.60 | (0.96 – 2.67) |  |
| Others* | 0.78 | (0.38 – 1.63) |  |
| Marital Status |  |  |  |
| Married | Reference | |  |
| Unmarried^+^ | 1.15 | (0.82 – 1.62) |  |
| Education |  |  |  |
| Some College or Technical School /  College Graduate | Reference | |  |
| Elementary / Some High School /  High School Graduate | 1.02 | (0.74 – 1.39) |  |
| Employment |  |  |  |
| Employed | Reference | |  |
| Unemployed^$^ | 0.61 | (0.27 – 1.35) |  |
| **Health Indicators** |  |  |  |
| General Health |  |  |  |
| Excellent / Very Good / Good | Reference | |  |
| Fair / Poor | **3.49** | **(2.49 – 4.86)** |  |
| Number of Chronic Conditions |  |  |  |
| 0 | Reference | |  |
| 1 | 1.08 | (0.59 – 1.97) |  |
| 2 | 1.32 | (0.75 – 2.35) |  |
| ≥ 3 | **1.78** | **(1.03 – 3.08)** |  |
| Smoking |  |  |  |
| No | Reference | |  |
| Yes | **1.75** | **(1.16 – 2.64)** |  |
| Use of Special Equipment due to a Health Problem |  |  |  |
| No | Reference | |  |
| Yes | **2.88** | **(2.11 – 3.93)** |  |
| **Health Care Indicators** |  |  |  |
| Have a Health Plan |  |  |  |
| Yes | Reference | |  |
| No | **0.36** | **(0.16 – 0.79)** |  |
| Have a Primary Care Provider |  |  |  |
| Yes | Reference | |  |
| No | 0.93 | (0.52 – 1.66) |  |
| Annual Health Checkup |  |  |  |
| Yes | Reference | |  |
| No | 1.10 | (0.71 – 1.69) |  |
|  |  |  |  |

* Includes non-Hispanic Asian, Native Hawaiian or other Pacific Islander,

American Indian or Alaskan Native only, Multiracial and Other race only.

+ Includes Divorced, Widowed, Separated, Never Married, and Member of an

unmarried couple.

$ Includes Homemaker, Student, Retired, Unable to Work, and Out of work.
